# Intrauterine death in vasa previa without hemorrhage: case reports

**DOI:** 10.1186/s12884-023-06019-0

**Published:** 2023-10-03

**Authors:** Pin Li, Xiuyu Pan, Chaomin Yue, Zheng Zheng, Huishu Liu

**Affiliations:** Department of Obstetrics, Guangzhou Women and Children’s Medical Center, Guangzhou Medical University, Guangzhou, 510623 China

**Keywords:** Vasa previa, Velamentous cord insertion, Intrauterine fetal death, Antepartum hemorrhage

## Abstract

Antepartum and intrapartum hemorrhage from vasa previa (VP) is one of the main causes of intrauterine fetal death (IUFD). Here, we present two cases with type I VP in which velamentous cord insertion below the fetal head and overlying the cervix were reported by prenatal ultrasound scanning, and IUFD occoured after 35 weeks with no signs of prenatal bleeding but with engaged fetal head at presentation. We hypothesized that the IUFD may attributed to the compression of the unprotected umbilical vessels by the engaged fetal head. Thus we suggest that VP with a velamentous cord insertion should be considered for earlier termination of the pregnancy to avoid the risk of non-hemorrhagic adverse fetal outcomes.

## Introduction

Intauterine fetal death (IUFD) is the end result of a variety of maternal, fetal, and placental disorders, which can interact to contribute to fetal demise [1]. In placental abnormalities, antepartum and intrapartum hemorrhage from vasa previa (VP) is one of the leading causes of IUFD. VP refers to a condition in which umbilical vessels, unprotected by either the umbilical cord or the placental tissue, traverse the fetal membranes of the lower uterine segment passing below the fetal presenting part [2–4]. After the onset of labor or rupture of membranes, these blood vessels overlying the cervix are easily torn, resulting in life-threatening, often fatal fetal hemorrhage [5, 6]. VP has been reported to occur in approximately 1 in 2,500 pregnancies and thus is considered one of the most dangerous obstetric complications with a high risk for perinatal mortality [4, 7]. IUFD with VP is usually caused by the fetal blood loss from rupture of unprotected placental vessels [8, 9]. However in the two cases we presented here, evidence of fetal blood loss were not found. While detrimental fetal, maternal or placental abnormalities were also not found prenatally or postnatally in these two cases, thrombosis were reported by pathologist in one of the case. At the same time, fetal head engagement were both found in the two cases. Thus occlusion of the VP was considered to be the cause of the IUFD in our two cases, which were described in details as follows.

## Case1

**Fig. 1:**
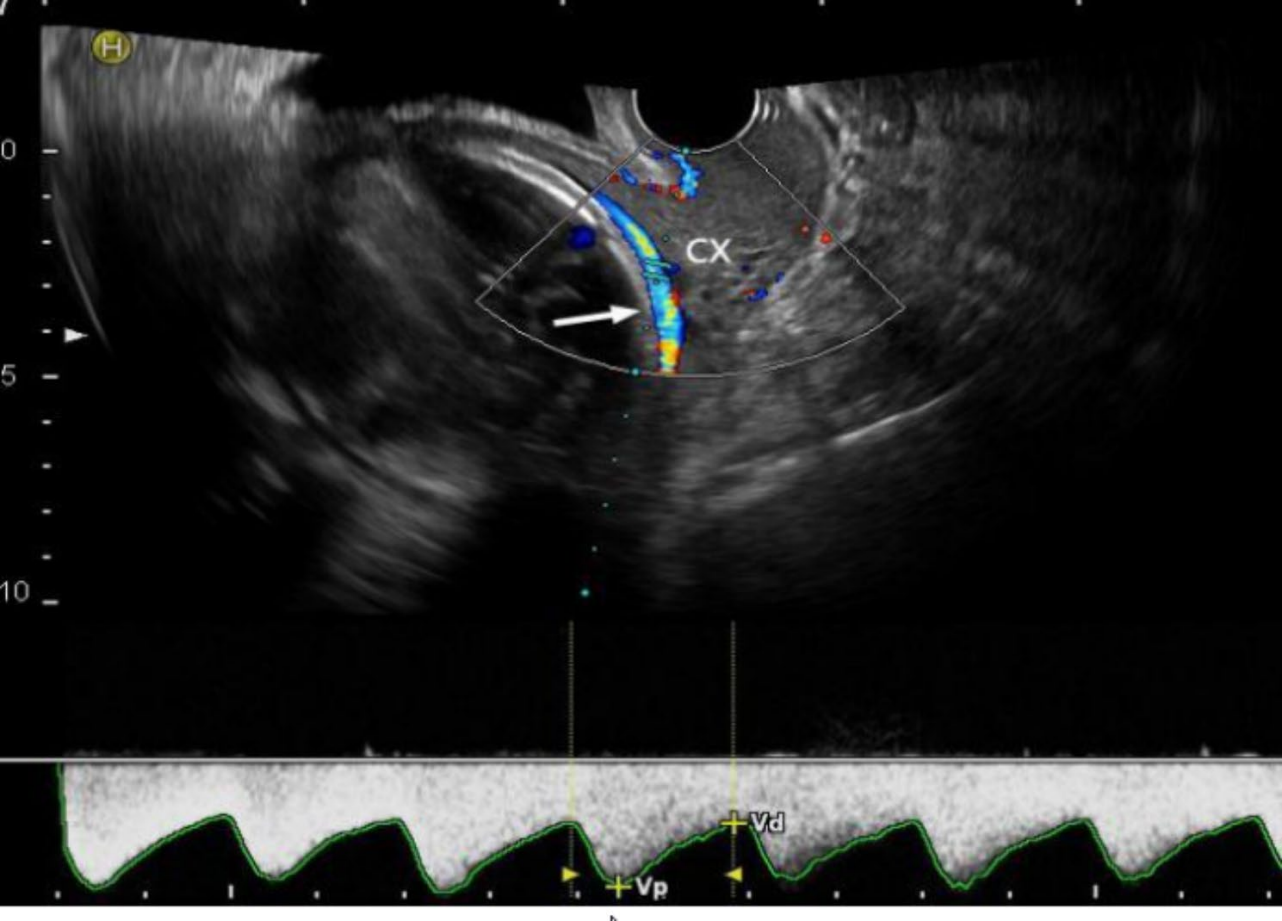
The umbilical cord insertion showed by sonographic scanning in case 1. White arrow indicates the umbilical insertion on the fetal membranes and overlay of the internal cervical os with arterial waveform observed above the internal cervical os at 32 weeks of gestation

**Fig. 2:**
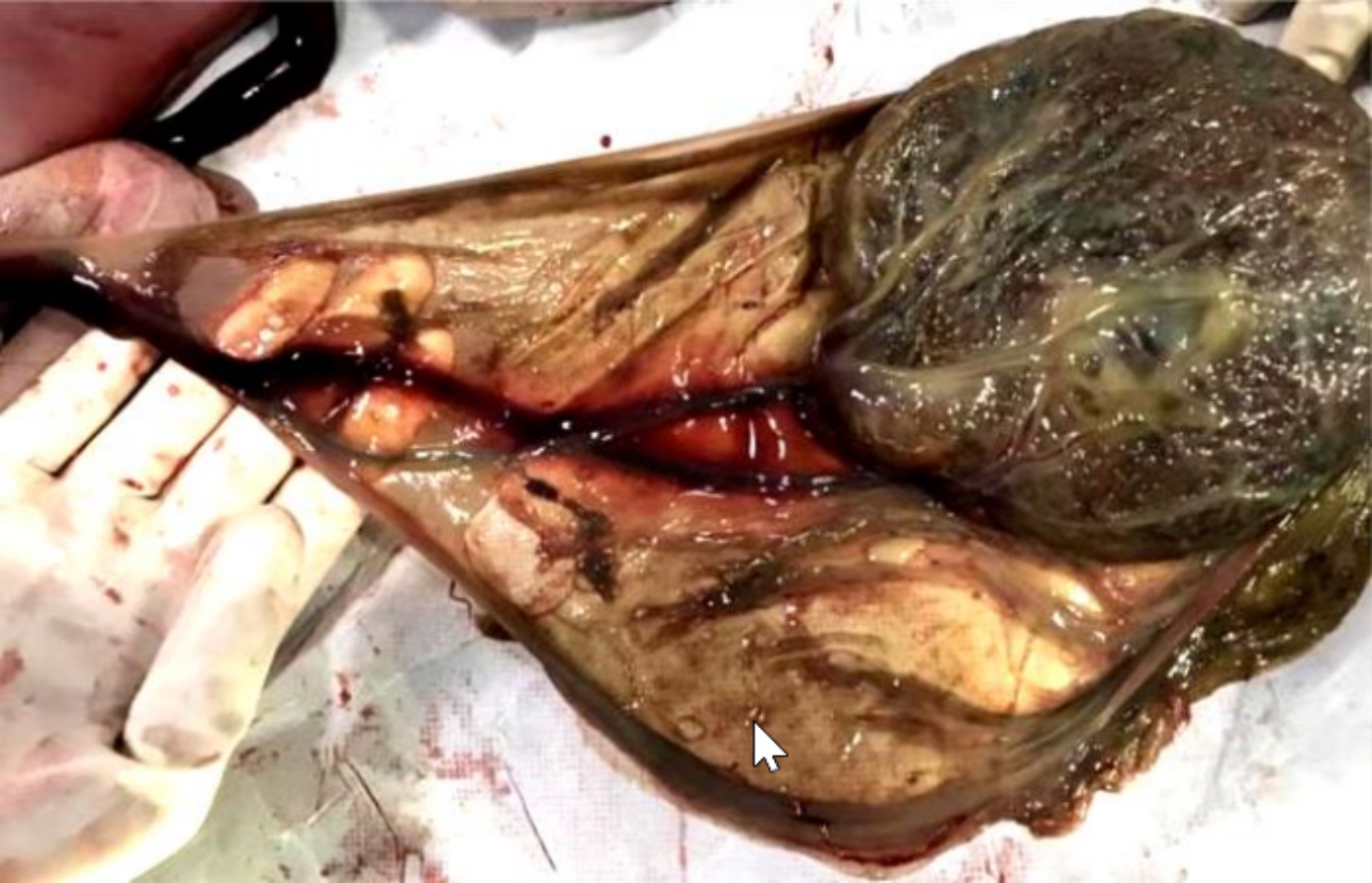
The umbilical insertion showed by gross examination in case 1. The velamentous cord insertion was about 15 cm from the margin of the thicker and smaller placenta, and the vessels running on the placental membrane observed

A 25-year-old female with gravida 4, para 2, abortion 1, was hospitalized at 35 + 5 weeks of gestation for a complaint of decreased fetal movement for 5 days and ultrasound confirmed IUFD one day. Her antenatal history was unremarkable except for a velamentous umbilical cord insertion and VP on mid-trimester transvaginal ultrasound. The third-trimester scan also demonstrated umbilical cord insertion external to the placenta and overlaying the internal cervical os with arterial and venous wave-forms observed above the internal cervical os (Fig. 1). She was advised to be hospitalized for antenatal corticosteroid therapy and close surveillance during 32–34 weeks gestation. However, she did not follow it. She denied any episodes of vaginal bleeding prior to presenting at 35 + 5 weeks. After hospitalization, induction with low-dose oxytocin was initiated, as the head was engaged and the Bishop score was 6. During induction, the umbilical vessels in the amniotic membrane were seen overlying the internal os through the dilated cervix. After a 10-hour induction, a normal-appearing fetus weighing 2,340 g was delivered. The velamentous cord insertion was about 15 cm from the margin of a somewhat thick and small placenta (Fig. 2). Suggestions for placental pathology and fetal autopsy was refused by the patient. She was discharged 24 h after delivery.

## Case2

A 34-year-old primigravida had routine prenatal checkups during pregnancy. She was healthy with no history of surgeries or diseases previously. She conceived natrually and underwent a smoth early pregnancy without any complications.

**Fig. 3:**
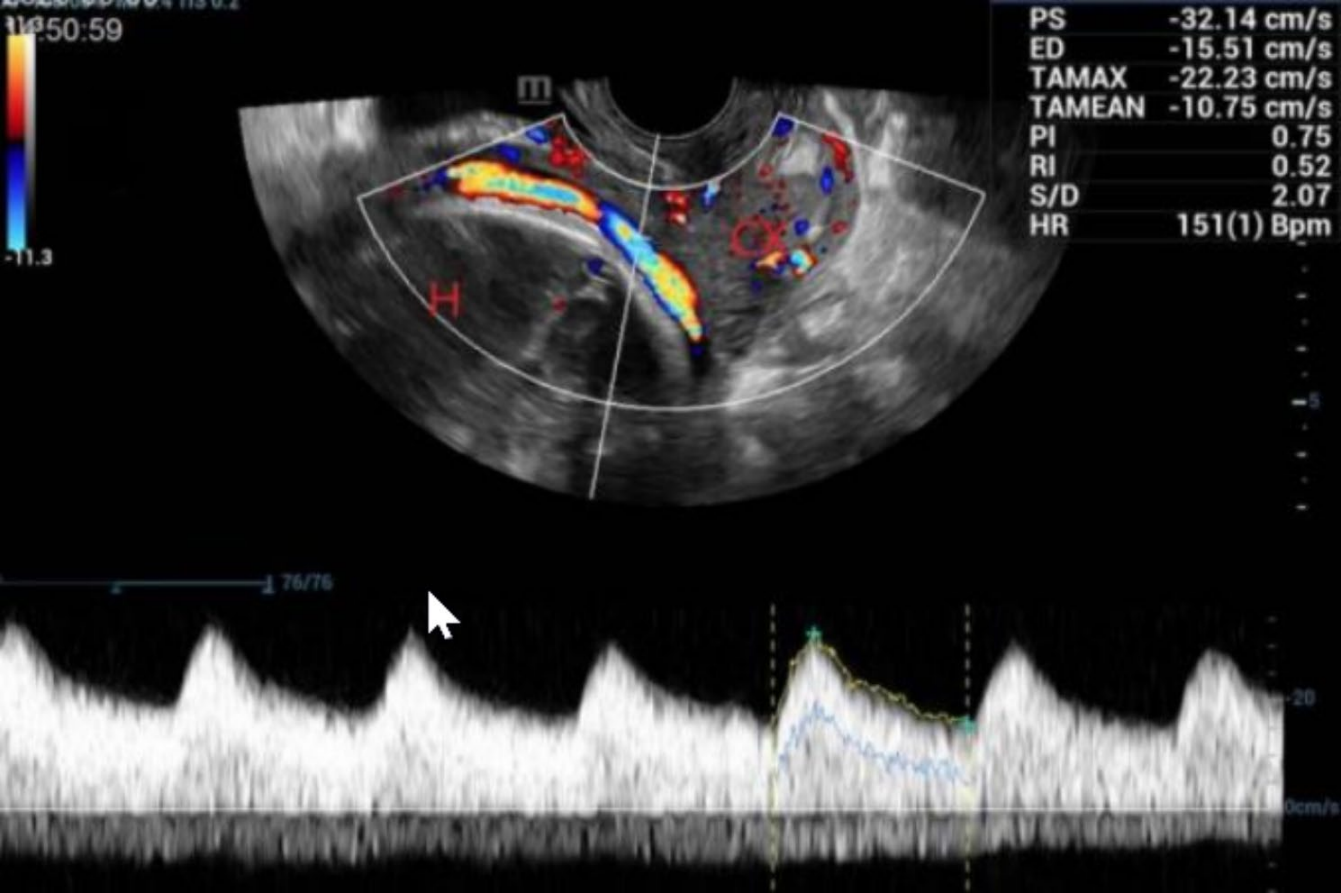
The fetal vessels showed by sonographic scanning in case 2. An arterial vessel and vein vessel were shown running under the fetal membrane, crossing the internal os to the anterior lower segment of the uterus at 33 weeks of gestation

She was referred to our hospital at 19 weeks for further assessment due to a suspected velamentous umbilical cord insertion and VP. Since she was asymptomatic, vaginal ultrasound with color and pulsed Doppler was arranged at 24 weeks to confirm the diagnosis. The ultrasound scanning reported that the umbilical cord insertion point located in the fetal membrane at the lower edge of the placenta, which was more than 2 cm away from the internal os. An arterial vessel and two veins were indicated to run in the fetal membranes, crossing the internal os to the anterior lower segment of the uterus (Fig. 3).

**Fig. 4:**
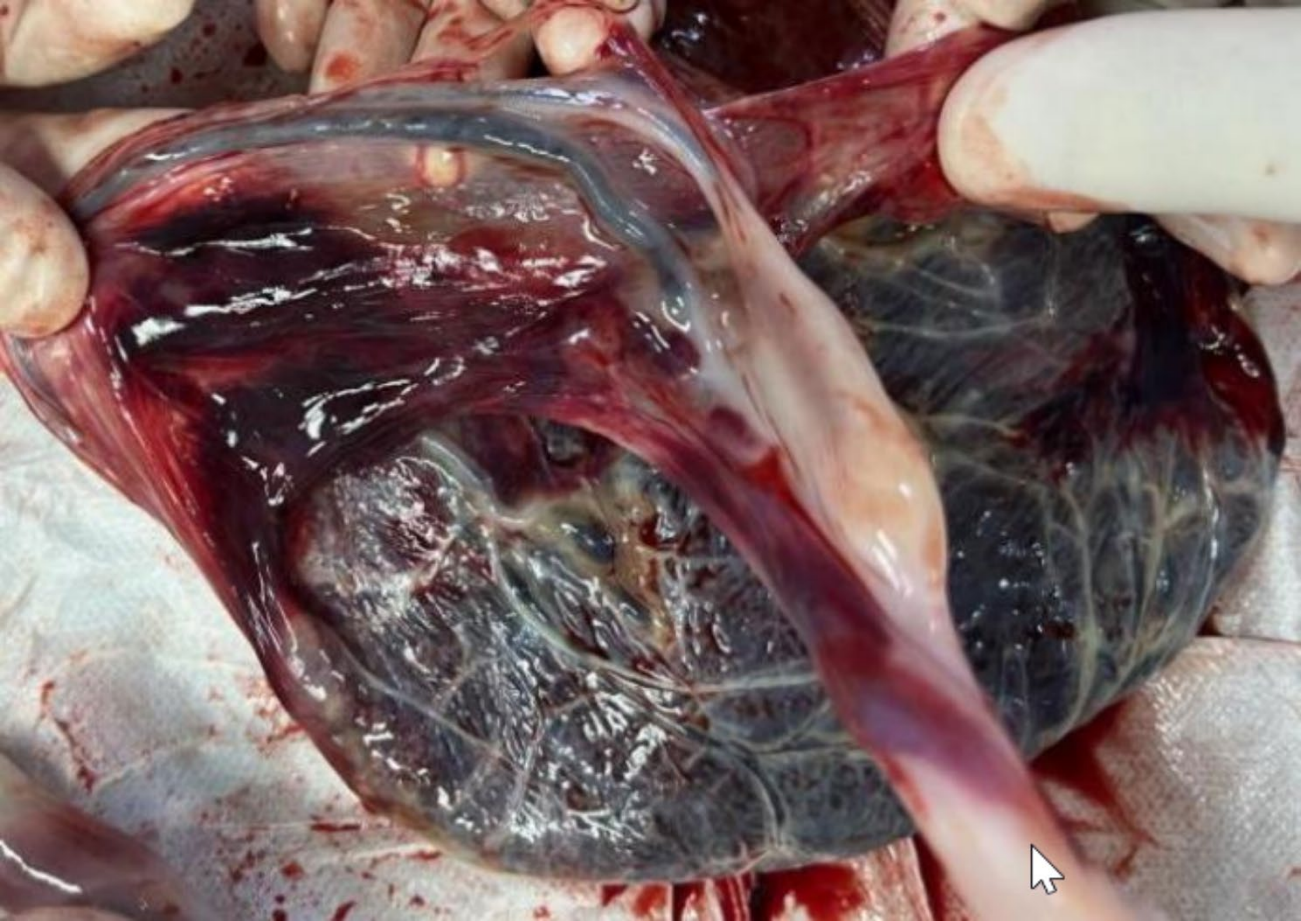
One major umbilical vessel running in the fetal membrane in case 2. One of the umbilical vessels could be observed crossing in the membranes and no obvious laceration of the velamentous vessels was observed

As diagnosis with VP, she had high possibility of early termination of the pregnancy. She was treated with antenatal corticosteroid at 32 weeks of gestation at outpatient clinic. She was advised to be hospitalization at 32 weeks and scheduled to have a cesarean delivery between 34 and 36 weeks but she refused. Fetal heart monitoring was performed weekly at outpatient clinic. She had no complaints of symptoms including threatened labor or vaginal bleeding before 36 + 6 weeks when she visited the hospital with complaint of no fetal movement. Fetal heart could not be detected by Doppler and IUFD was confirmed by ultrasound. Though the head was engaged, the Bishop score was unfavorable. She received a Foley balloon for cervical ripening and after that, Low-dose oxytocin was administrated for labor induction. As contractions commenced, she experienced minor vaginal bleeding. She delivered a fetus weighing 2,680 g after 12 hours’ labor. The appearance of the fetus was grossly normal. The umbilical cord was 60 cm and wrapped around one of the hands loosely for a circle. The umbilical vessels were observed crossing in the fetal membranes and no obvious laceration of the velamentous vessels was observed (Fig. 4). She was discharged without any complications after delivery. Placental pathology showed thrombosis and inflammation in fetal and chorionic vessels.

## Discussion

VP is usually classified into three types: type I (the umbilical vessels connect a velamentous umbilical cord to a morphologically normal placenta, type II (the umbilical vessels insert into one lobe of a bilobed placenta or a succenturiate lobe) [9, 10] and some rare cases of type III (the unprotected fetal vessels running outside of the placenta across or near the internal os with a normal umbilical cord insertion) [7, 11]. Type I accounts for about 76.6-89.5% of VP while 10.5-14.1% of them could be categorized as Type II [12, 13]. Our two cases could be classified as Type I VP but with some specific common characteristics: (a) velamentous cord insertion below the fetal head and overlying the cervix; (b) gestational age of 35–36 weeks at the time of IUFD; (c) engaged fetal head at presentation when IUFD occurs; (d) no history of apparent vaginal bleeding.

IUFD is the end result of a variety of maternal, fetal, or placental disorders [1]. Antepartum or intra-partum hemorrhage is a well-known cause of IUFD for the pregnancies with VP. However, both the cases presented here didn’t report any detectable prenatal bleeding before IUFD. In our cases, both the patients were healthy with no history of major health problems or any pregnant complications. Though no autopsy was performed, the normal prenatal screening, grossly normal appearance and normal range birth weights disproved the possiblity of fetus-related IUFD. In our cases of IUFD, the maternal or fetal risks were primarily excluded. The thicker and smaller placenta in case 1 and wrapped umbilical cord with hand for one circle in case 2 were not sufficient to explain the IUFD. Thrombosis found in chorionic vessels indicated the possible reason of the IUFD: disturbance of fetal circulation. Combined with the shared characteristics of these two cases, we presume that the occurrence of IUFD for our cases might be caused by the cease of fetal circulation due to the compression of unprotected fetal vessels by the presenting part.

Though, VP is conventionally defined as fetal blood vessels running in the membranes within 2 cm from the internal os of cervix [14], under the assumption of the causes of IUFD in our cases, we should also pay attention to those cases within 5 cm from cervix, which was also called for by Dr.Oyelese [15]. Our logic is like this: assuming the fetus’s biparietal diameter is 8–10 cm in the third-trimester, fetal vessels which lie within 4–5 cm of the internal os may be compressed by the descending fetal head during engagement. Therefore, the presence of VP less than 5 cm from the internal os should be considered potentially dangerous and alternative/early delivery plans should be recommended before tight engagement, which usually happens with threaten labor or premature rupture of membrane.

With the advances in prenatal diagnosis, closer monitoring and appropriate termination of the pregnancy, the perinatal mortality rate of VP is less than 10% [16–19]. Some guidelines recommended that the optimal range of gestational age for delivery was from 34 to 37 weeks [4]. According to guidelines and our clinical experience, we recommend the following strategies for prenatal diagnosed VP: a). close fetal heart monitoring should be conducted from 30 to 32 weeks, which is more accessible with popularity of remote fetal monitoring devices and artificial intelligence interpretation; b). fetal lung maturity should be launched during 32 weeks; c). planned cesarean delivery at 34–36 weeks of gestation in asymptomatic women. Our previous retrospective analysis of 116 cases with VP confirmed that: prophylactic hospitalization before 32 weeks of pregnancy, and close monitoring of premature birth signs before 34 weeks of gestation improved the perinatal outcomes [13]. With the lessons from the two IUFDs, we suggest that velamentous cord insertion site below the fetal presentation need timely termination of pregnancy before fetal presentation engaged with or without threaten preterm labor to avoid adverse outcomes.

In summary, fetal demise in pregnancies with VP is not just caused by bleeding. IUFD may also be caused by anoxia secondary to vessel compression by the presenting part. For pregnancies with velamentous cord insertion, we recommend that the distance from the internal os should be carefully reported by mid-trimester ultrasonography and fetal vessels within 5 cm also should bemanaged as VP. In the case of Type I VP, in which velamentous cord insertion site below the fetal presentation and big (major) fetal vessels overlying the cervix, we recommend that pregnancy should be terminated before fetal presentation engagement with or without threaten preterm laboring.

## Data Availability

Only available from the corresponding author on reasonable request.
